# Association between equine asthma and fungal elements in the tracheal wash: An environment-matched case-control study

**DOI:** 10.1371/journal.pone.0309835

**Published:** 2024-09-06

**Authors:** Sarah Dély, Vinzenz Gerber, Laureen M. Peters, Sophie E. Sage

**Affiliations:** 1 Vetsuisse Faculty, Department of Clinical Veterinary Medicine, Swiss Institute of Equine Medicine (ISME), University of Bern, Bern, Switzerland; 2 Vetsuisse Faculty, Department of Clinical Veterinary Medicine, Clinical Diagnostic Laboratory, University of Bern, Bern, Switzerland; Children’s National Hospital, George Washington University, UNITED STATES OF AMERICA

## Abstract

The presence of fungi in tracheal wash (TW) of horses was recently linked to mild-moderate equine asthma, indicating a possible causal role; however, increased numbers of fungi may also stem from asthma-related alteration of tracheal mucus clearance or from environmental exposure. Our objective was to elucidate the association between the presence of fungi in TW and asthma status while controlling for relevant confounders. We conducted a retrospective case-control study involving 73 horses, including 34 controls and 39 asthmatic cases. Each asthmatic horse was matched with a control from the same barn to account for the influence of environmental exposure. All horses underwent respiratory clinical scoring, endoscopy, TW, and bronchoalveolar lavage (BAL). The association between asthma status and presence of TW fungi was tested with multivariable logistic regression modelling, accounting for selected management factors, tracheal mucus accumulation, and selected TW and BAL cytological characteristics, including multinucleated giant cells (MGCs) in the TW. Given the variability in MGC definitions in the literature, particularly concerning their morphology and number of nuclei, we constructed two distinct models for each outcome (asthma status or presence of fungi in TW): one considering MGCs as cells with ≥ 3 nuclei, and another using a criterion of ≥ 10 nuclei. Horses with a tracheal mucus score ≥ 2 exhibited 3.6 to 4.3 higher odds of being asthmatic, depending on the MGC definition. None of the other variables examined were associated with either asthma status or TW fungi detection. Notably, the presence of fungal elements in the TW was not associated with equine asthma.

## Introduction

Equine asthma is a common respiratory disease and an important cause of reduced performance [1]. The exact pathophysiology of the disease is yet unknown, but asthma can be triggered by inhaled particles found in environmental dust, including organic components, such as endotoxins, β-glucans, and allergenic proteins from fungal elements [2]. A link between inflammatory airway disease (now called mild-moderate equine asthma) and the presence of fungal particles in the lower airways was recently suggested [3]. In that study, horses with fungal elements identified on tracheal wash (TW) cytology were twice as likely to be diagnosed with mild-moderate equine asthma. In addition, growing fungi (branching hyphae and germinating spores) were observed in TW and bronchoalveolar lavage fluid (BALF). The fungi isolated from TW mycological cultures in the study by Dauvillier et al. [3] included *Penicillium* (53%), *Aspergillus* (34%), *Rhizomucor* (5%), and *Candida* (5%). However, the agreement between mycological culture results and fungal detection on cytology was poor, making it difficult to determine the specific fungal species associated with equine asthma based on cytology alone. The authors proposed that inhaled fungi may contribute to the pathophysiology of equine asthma, similarly to what is seen in fungi-related asthma or in allergic broncho-pulmonary aspergillosis (ABPA) in human patients [4–6].

Fungal elements are ubiquitous in the environment of horses [7] and encompass spores, hyphae, or fragments of these structures. Their detection indicates exposure to fungi but does not confirm an active infection. Besides *Aspergillus fumigatus*, several other fungal genera have been identified in equine pulmonary samples such as *Aspergillus* (species other than *A*. *fumigatus*), *Penicillium*, *Alternaria*, *Mucor*, and *Rhizopus* [3,8–12]. However, the species detected vary enormously from one study to another and depend on multiple factors, such as the location of sampling within the respiratory tract [10] or the season [12]. Methods for identifying fungal elements in TW include light microscopy, mycological cultures and PCR assays targeting fungal DNA, with the latter two providing more precise species identification. The pathogenic potential of fungal elements can be exerted by triggering an allergic response, through colonization of the airways, or a combination of both [13]. In asthmatic patients, fungi are thought to act mainly as a source of allergic sensitization, eliciting a Th2-type response [6,14–16]. This hypothesis was further substantiated by two recent studies which found increased concentrations of fungi-specific immunoglobulin E (IgE) in both serum and BALF of asthmatic horses [17,18]. Furthermore, previous studies identified recombinant *Aspergillus fumigatus* allergen-specific IgE levels as a biomarker for asthma in horses [19,20] and ABPA in humans [21,22]. Fungal colonization and growth in the airways typically leads to a greater immune response in the host than sole allergenic agents as described in human medicine for ABPA and fungi-related severe asthma [4,6].

The link between the presence of fungal elements in the airways and the development of asthma in horses remains unclear. Although Dauvillier and colleagues [3] reported an association between TW fungal elements and mild-moderate equine asthma, a recent study found fewer fungi in the BALF from stabled asthmatic horses compared to controls [9]. This discrepancy could stem from differences in study design and sample types, with TW fungal load possibly influenced by environmental exposure [8]. Furthermore, increased detection of fungi in the TW of asthmatic horses may reflect compromised tracheal mucus clearance associated with the disease [1,23].

Recent findings also suggest a potential link between multinucleated giant cells (MGCs) in BALF and equine asthma [24], consistent with observations in human chronic lung diseases [25]. These MGCs are believed to result from the fusion of multiple macrophages in response to persistent microorganisms or materials [26], contributing to granuloma formation, as observed in horses with idiopathic granulomatous pneumonia [27]. Therefore, we explored the presence of MGCs in TW to investigate their potential link with equine asthma and fungi detection. Given the inconsistent definitions of MGCs in the literature [24–26,28–30], we examined MGCs in TW considering either MGC_3_ (cells with ≥ 3 nuclei) [26,29] or MGC_10_ (cells with ≥ 10 nuclei) [25].

Hence, this study aims to clarify the relationship between the asthma status of horses and the detection of fungi in TW samples while accounting for selected management factors, tracheal mucus accumulation and selected TW and BAL cytological characteristics, including TW MGCs.

## Material and methods

### Study design and population

This retrospective controlled study comprised a population of 83 horses (39 controls and 44 asthmatic cases) included in a previous study from our group [18]. Asthmatic horses were recruited through referring veterinarians and by advertising on social media. Suitable candidates were identified using the horse-owner assessed respiratory signs index (HOARSI) [31]. Horses with a HOARSI > 1 were invited to the clinic for potential inclusion as asthmatic cases. The horse owners were asked to bring another horse from the same barn without a history of respiratory signs or a prior asthma diagnosis. A HOARSI questionnaire, including questions on feeding and management practices, was filled for the control horse upon admission to the clinic. All horses underwent a complete respiratory work-up, including clinical score [32], endoscopy, tracheal mucus score [33], TW, and BAL. The main results are listed in Table 1. For a detailed description of sample collection and processing (except for TW, which is outlined below), readers are directed to the study by Wyler et al. [18]. The study protocol was approved by the Animal Experimentation Committee of the Canton of Bern, Switzerland (BE4/20+). Informed consent was obtained from the owners of all horses included in the study.

**Table 1:** Description of the horse population.

Characteristic	Control, N = 34^1^	Asthmatic, N = 39^1^	Likelihood ratio test *P*-value
Age (years)	12 (9, 18)	14 (9, 18)	0.69
Weight (kg)	554 (525, 623)	514 (419, 564)	NA (unequal groups)
Sex			0.98
Mare	13 (38%)	15 (38%)	
Gelding	20 (59%)	22 (56%)	
Stallion	1 (3%)	2 (5%)	
HOARSI (/4)			**< 0.001**
1	24 (71%)	0 (0%)	
2	8 (24%)	3 (8%)	
3	2 (6%)	4 (10%)	
4	0 (0%)	32 (82%)	
Clinical score (/23)	1 (0, 2)	5 (2, 7)	**< 0.001**
Tracheal mucous score (/5)			**< 0.01**
0	7 (21%)	3 (8%)	
1	10 (29%)	4 (10%)	
2	13 (38%)	12 (31%)	
3	4 (12%)	7 (18%)	
4	0 (0%)	11 (28%)	
5	0 (0%)	2 (5%)	
BALF yield (%)	56 (45, 60)	52 (40, 60)	0.57
BALF macrophages (%)	40.0 (30.6, 46.6)	33.0 (25.1, 43.5)	0.25
BALF lymphocytes (%)	49.9 (44.6, 54.9)	50.0 (36.4, 57.5)	0.40
BALF neutrophils (%)	8.4 (5.6, 12.8)	12.8 (6.2, 22.5)	**0.02**
BALF mast cells (%)	1.4 (0.6, 2.2)	2.0 (0.6, 2.9)	0.14
BALF eosinophils (%)	0.0 (0.0, 0.4)	0.0 (0.0, 0.3)	0.62

NA: Not available (missing value).

^1^Median (IQR); n (%).

### Collection, processing, and cytological examination of TW samples

TW sample collection was achieved by instillation of 10 ml of sterile 0.9% saline solution through a Teflon-coated PVC catheter inserted through the endoscope (VET-OR1200HD, Medical Solution GMBH, Wil, Switzerland) channel and direct reaspiration of the saline mixed with secretions. Direct smears were prepared immediately after collection and rapidly air-dried with a cold hair dryer. The direct smears were then stained within 48h with modified Wright-Giemsa stain and stored for future analyses.

One stained direct smear of TW was retrieved for each horse included in the present study. One author (SD) performed an initial cytological analysis of a training set consisting of 15 direct smears of TW randomly selected (10 cases and 5 controls). The first 5 direct smears were reviewed by SD together with a board-certified clinical pathologist (LP). The next 10 direct smears were first reviewed separately by SD and LP, results were then compared, and discrepancies were clarified. A scoring system was established (see below). Once the training was completed, the labels of the 15 direct smears were restored, ensuring independent re-evaluation of these as part of the sample set without being biased by prior results. Both SD and LP were blinded to the origin of the direct smears. All direct smears were examined by SD, and LP was consulted to review the scoring where uncertainties arose.

To account for heterogeneous cell distribution, the entire stained area of the smears was examined in a serpentine pattern at 100x or 200x magnification. Five areas were selected based on adequate cellularity, thickness, cell distribution, and staining quality for observation at higher magnification (500x and 1000x oil immersion). The quality of each direct smear was assessed with a semi-quantitative score for overall cellularity, mucus, Curschmann’s spirals, epithelial cells, and contamination with debris (0: none or rare; 1: mild; 2: moderate; 3: high). Direct smears presenting a cellularity score of 0 were excluded. The same semi-quantitative score was assigned to neutrophils, lymphocytes, eosinophils, mast cells, macrophages, and MGCs, whereby the latter were assigned one score for MGCs with ≥ 3 nuclei (TW MGC_3_) and one score for MGCs with ≥ 10 nuclei (TW MGC_10_) [25,26,29]. Fungal elements, including spores, conidia, conidiophores, hyphae, and mycelia, were recorded in the same manner, and presence or absence of intracellular fungal elements, spore germination, branching hyphae, and bacteria was specifically noted. A description of each fungal element identified was also provided.

### Statistical analyses

The association between the asthma status of the horse and the presence of fungal elements in the TW was assessed using logistic regression modelling. To improve the performance of the statistical models, the categorical variables with multiple levels were transformed into binary variables (see S1 Table). Special care was taken to limit the number of predictor variables included to avoid overfitting: only the variables deemed biologically relevant to assess the association between the detection of TW fungal elements and the asthma status of horses were considered. For example, the feeding type was included because feeding dry hay can increase exposure to fungal particles [3]. Variables with no or low number of observations per level were excluded (semi-quantitative scores for lymphocytes, eosinophils and mast cells in the TW, presence/absence of intracellular fungal elements, spore germination, branching hyphae or bacteria in the TW).

We first built a logistic regression model considering the presence of fungal elements in the TW as the outcome variable. The predictor variables considered for inclusion in the model were asthma status, age, sex, feeding type, bedding type, tracheal mucus score, presence of Curschmann’s spirals in TW, TW scores for neutrophils, TW MGC_3_, and BALF neutrophil percentage. An alternative model was constructed using TW MGC_10_ as a substitute variable for TW MGC_3_.

A second model was built considering the asthma status (case vs control) as the outcome variable. The predictor variables considered for inclusion in the model were age, sex, feeding type, bedding type, tracheal mucus score, presence of fungal elements in TW, presence of Curschmann’s spirals in TW, TW scores for neutrophils, TW MGC_3_, and BALF neutrophil percentage. An alternative model was constructed using TW MGC_10_ as a substitute variable for TW MGC_3_.

For both analyses, predictor variables with a likelihood test ratio *P*-value < 0.20 were selected for entry in the multivariable model, at the exception of the variables “asthma status” in the first model and “presence of fungal elements in the TW” in the second model, where the P-value was 0.28 (Tables 2 and 3). These variables were included in the multivariable model to ensure comprehensive examination of their potential association with the outcome of interest, aligning with the primary objective of the study. The impact of outliers on the model’s coefficients was tested. We made sure that removal of variables from the model did not change the variables coefficient by more than 20%. Interaction between predictor variables were tested. For each of the two outcomes considered, the best model was selected based on the lowest AIC score. Significance for the final logistic regression models was set at *P-*value < 0.05.

**Table 2:** Analysis of the association between the presence of fungal elements in TW of horses and selected predictor variables.

Variable	Absence of TW fungi^1^	Presence of TW fungi ^1^	Likelihood ratio test *P*-value	Selected for multivariable modelling
Presented as			0.28	Yes
Control	14 (40%)	20 (53%)		
Asthmatic	21 (60%)	18 (47%)		
Age (years)	15 (9, 20)	13 (9, 16)	0.40	No
Sex			0.78	No
Female	14 (40%)	14 (37%)		
Male	21 (60%)	24 (63%)		
Feeding type			**0.13**	**Yes**
Dry hay	22 (63%)	30 (79%)		
Low-dust	13 (37%)	8 (21%)		
Bedding type			0.76	No
Straw	19 (54%)	22 (58%)		
Low-dust	16 (46%)	16 (42%)		
Tracheal mucous score (/5)			0.21	No
[0 – 1]	9 (26%)	15 (39%)		
[2 – 5]	26 (74%)	23 (61%)		
Curschmann’s spiral			0.46	No
Absence	12 (34%)	10 (26%)		
Presence	23 (66%)	28 (74%)		
TW neutrophil			0.54	No
No to rare	15 (43%)	19 (50%)		
Moderate to high	20 (57%)	19 (50%)		
TW MGC_3_			**0.03**	**Yes**
Absence	20 (57%)	12 (32%)		
Presence	15 (43%)	26 (68%)		
TW MGC_10_			**0.02**	**Yes**
Absence	28 (80%)	21 (55%)		
Presence	7 (20%)	17 (45%)		
BALF neutrophil (%)	8.2 (4.6, 13.5)	10.9 (6.1, 19.9)	**0.04**	**Yes**

^1^Median (IQR); n (%).

*TW*, *tracheal wash; MGC*_*3*_, *multinucleated giant cells with ≥ 3 nuclei; MGC*_*10*_, *multinucleated giant cells with ≥ 10 nuclei*.

**Table 3:** Analysis of the association between asthma status of horses and selected predictor variables.

Variable	Control, N = 34^1^	Asthmatic, N = 39^1^	Likelihood ratio test *P*-value	Selected for multivariable modelling
Age (years)	12 (9, 18)	14 (9, 18)	0.69	No
Sex			0.98	No
Female	13 (38%)	15 (38%)		
Male	21 (62%)	24 (62%)		
Feeding type			**0.05**	**Yes**
Dry hay	28 (82%)	24 (62%)		
Low-dust	6 (18%)	15 (38%)		
Bedding type			0.96	No
Straw	19 (56%)	22 (56%)		
Low-dust	15 (44%)	17 (44%)		
Tracheal mucous score (/5)			**< 0.01**	**Yes**
[0 – 1]	17 (50%)	7 (18%)		
[2 – 5]	17 (50%)	32 (82%)		
TW fungi			0.28	Yes
Absence	14 (41%)	21 (54%)		
Presence	20 (59%)	18 (46%)		
Curschmann’s spiral			0.37	No
Absence	12 (35%)	10 (26%)		
Presence	22 (65%)	29 (74%)		
TW neutrophil			**< 0.05**	**Yes**
No to rare	20 (59%)	14 (36%)		
Moderate to high	14 (41%)	25 (64%)		
TW MGC_3_			0.37	No
Absence	13 (38%)	19 (49%)		
Presence	21 (62%)	20 (51%)		
TW MGC_10_			**0.16**	**Yes**
Absence	20 (59%)	29 (74%)		
Presence	14 (41%)	10 (26%)		
BALF neutrophil (%)	8.4 (5.6, 12.8)	12.8 (6.2, 22.5)	**< 0.02**	**Yes**

^1^Median (IQR); n (%).

*TW*, *tracheal wash; MGC*_*3*_, *multinucleated giant cells with ≥ 3 nuclei; MGC*_*10*_, *multinucleated giant cells with ≥ 10 nuclei*.

## Results

### Study population

The direct smears of TW of 83 horses were examined, of which 5 horses had missing or poor-quality smears. Therefore, 10 horses (5 environmentally matched pairs) were excluded. A total of 73 horses (34 controls, 39 asthmatics) were included in the final analysis. These horses came from 34 different barns, with a 1:1 matching ratio, except for 4 control horses matched with several asthmatic horses from the same barn (3 controls with 2 cases, 1 control with 3 cases). All raw data associated with this study are available in S2 Table.

The median age was 13 years (5–29 years). The represented breeds were Warmblood (n = 36), American Quarter Horse (n = 7), Freiberger (n = 5), Icelandic horse (n = 6), Welsh Cob (n = 5), Arabian (n = 3), Purebred Spanish Horse (n = 3), Haflinger (n = 2), Lusitano (n = 2), Shetland Pony (n = 2), and Standardbred (n = 2). The studied population comprised 42 geldings, 28 mares and 3 stallions. The complete description of the study population can be found in Table 1. The HOARSI, clinical score, tracheal mucus score and BALF neutrophil count were significantly higher in asthmatic horses compared to controls.

### Environmental exposure

Most horses (n = 52; 71.2%) were fed dry hay and/or straw, while 21 horses (28.8%) horses were fed low-dust alternatives (wet hay, haylage, steamed hay, or hay cubes). Forty-one horses (56.2%) were bedded on straw, compared to 32 horses (43.8%) bedded on a low-dust alternative (sawdust, shavings, hemp, forest floor, straw pellets, rubber mat or other). Some pairs of horses had mismatched feeding or bedding type, despite being housed in the same barn. Specifically, 8 asthmatic horses were fed low-dust alternatives, while their matched controls were fed hay and/or straw. Seven horses (4 controls and 3 asthmatics) were bedded on low-dust alternatives while their paired counterparts were bedded on straw. Feeding type, but not bedding type, was significantly different between the asthmatic and control groups, with asthmatic horses more likely to be fed low-dust forages.

The results of the univariable analysis for each predictor variable can be found in Table 2 (outcome: presence of fungal elements in the TW) and Table 3 (outcome: asthma status).

### TW samples characteristics

**Fig 1:**
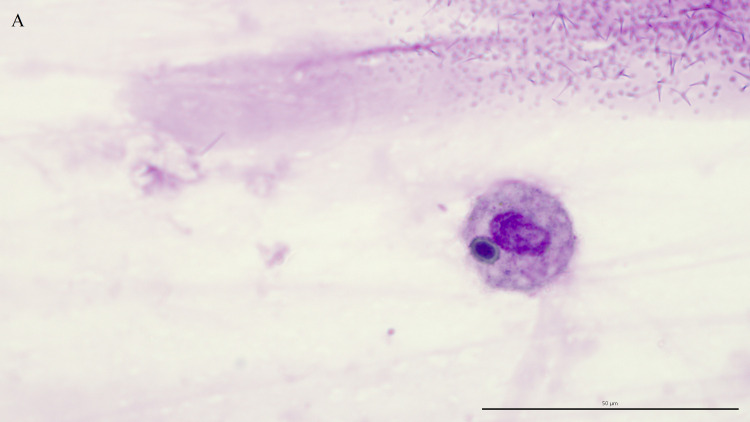
**Macrophage with an intracellular spore.** Direct smear of tracheal wash stained with modified Wright-Giemsa stain at 1000x oil magnification, bar = 50 μm.

**Fig 2:**
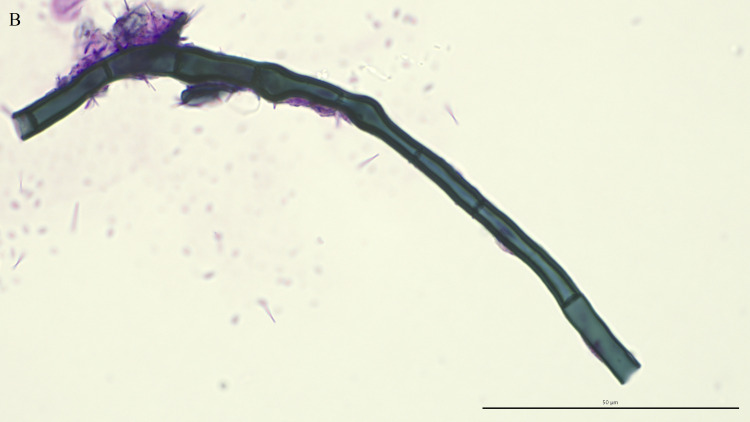
Fungal hyphae. Direct smear of tracheal wash stained with modified Wright-Giemsa stain at 1000x oil magnification, bar = 50 μm.

**Fig 3:**
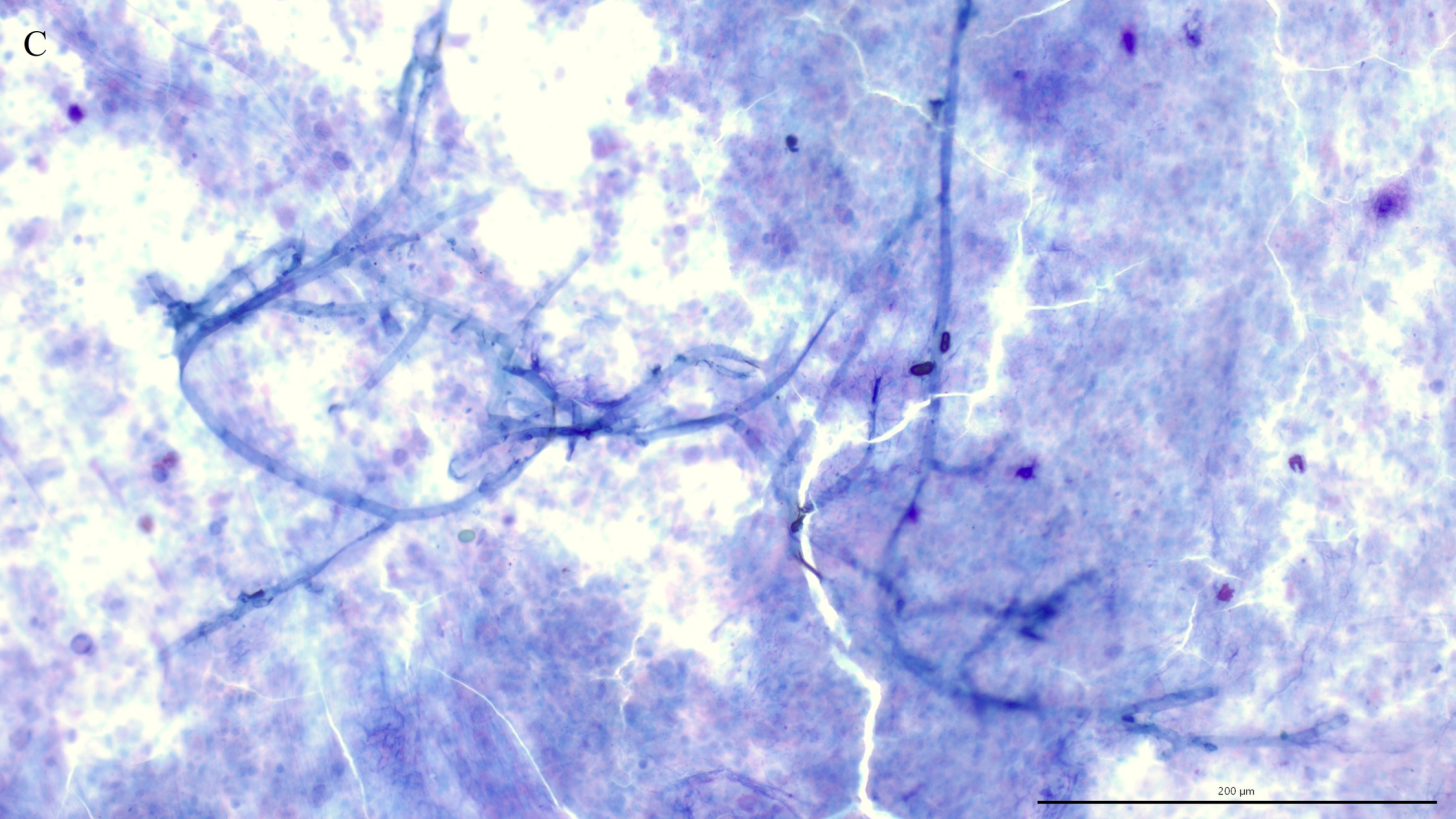
Macrophage with an intracellular spore. Direct smear of tracheal wash stained with modified Wright-Giemsa stain at 200x magnification, bar = 200 μm.

Fungal elements were present in 38 (52%) of the 73 TW direct smears examined. A third of these smears (13/38, 34%) contained intracellular fungi (Fig 1), including 9 controls and 4 asthmatics. Fungal hyphae (Fig 2), identified on 36 TW direct smears, were the most frequently detected fungal elements. The majority of fungal elements appeared as septate hyphae with acute angle branching, suggestive of *Aspergillus* spp. or similar, with short, bottle-shaped hyphae, consistent with *Alternaria* spp., observed in 8 cases. The remaining two direct smears contained only intracellular spores. Branching hyphae (Fig 3) were identified on only 3 TW direct smears, 2 of which were from control horses.

Bacteria were detected on 3 direct smears (2 controls, 1 asthmatic), with intracellular bacteria exclusively found in the smear of the asthmatic horse.

**Fig 4:**
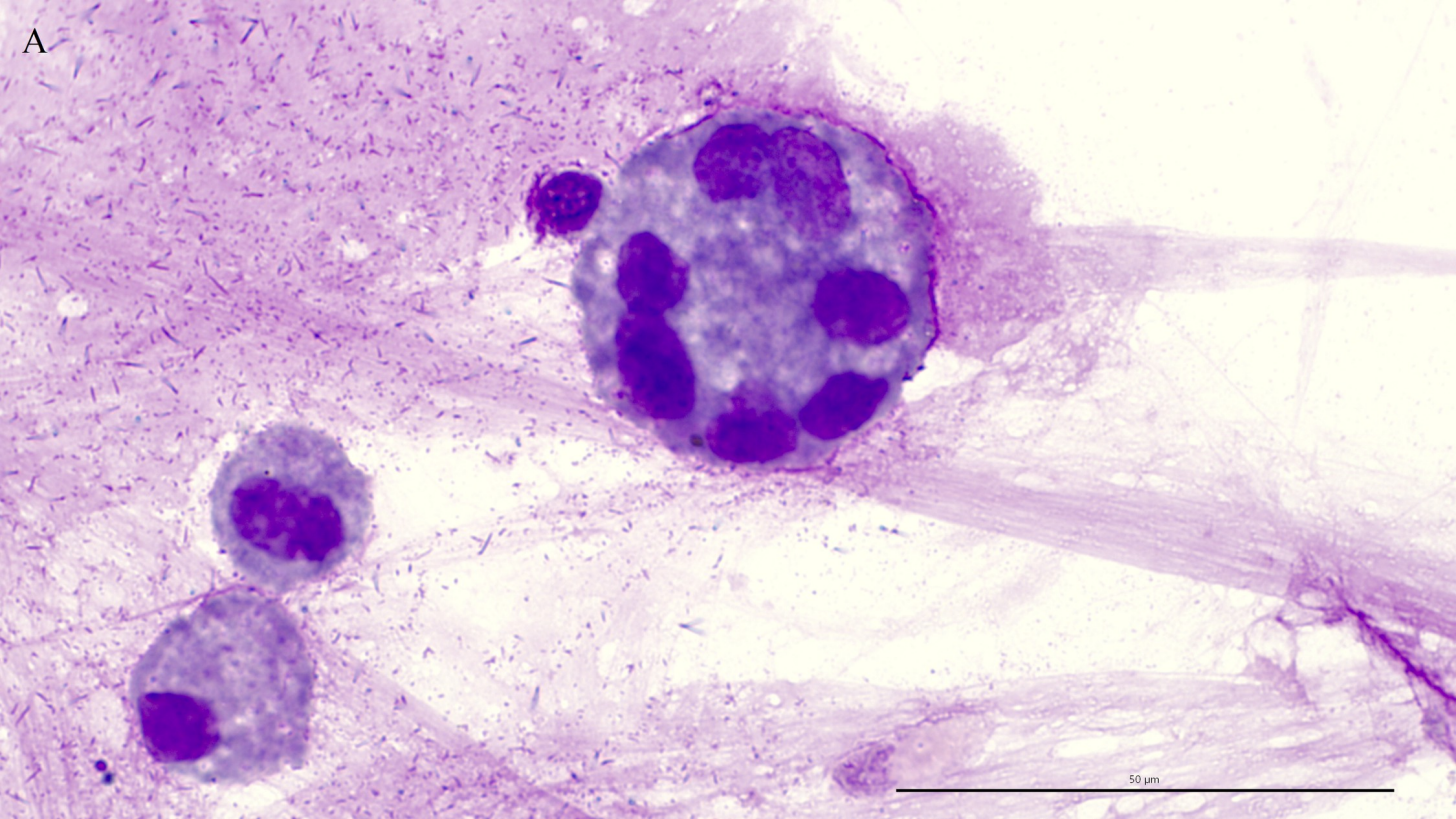
Multinucleated giant cell with ≥ 3 nuclei. Direct smear of tracheal wash stained with modified Wright-Giemsa stain at 1000x oil magnification, bar = 50 μm.

**Fig 5:**
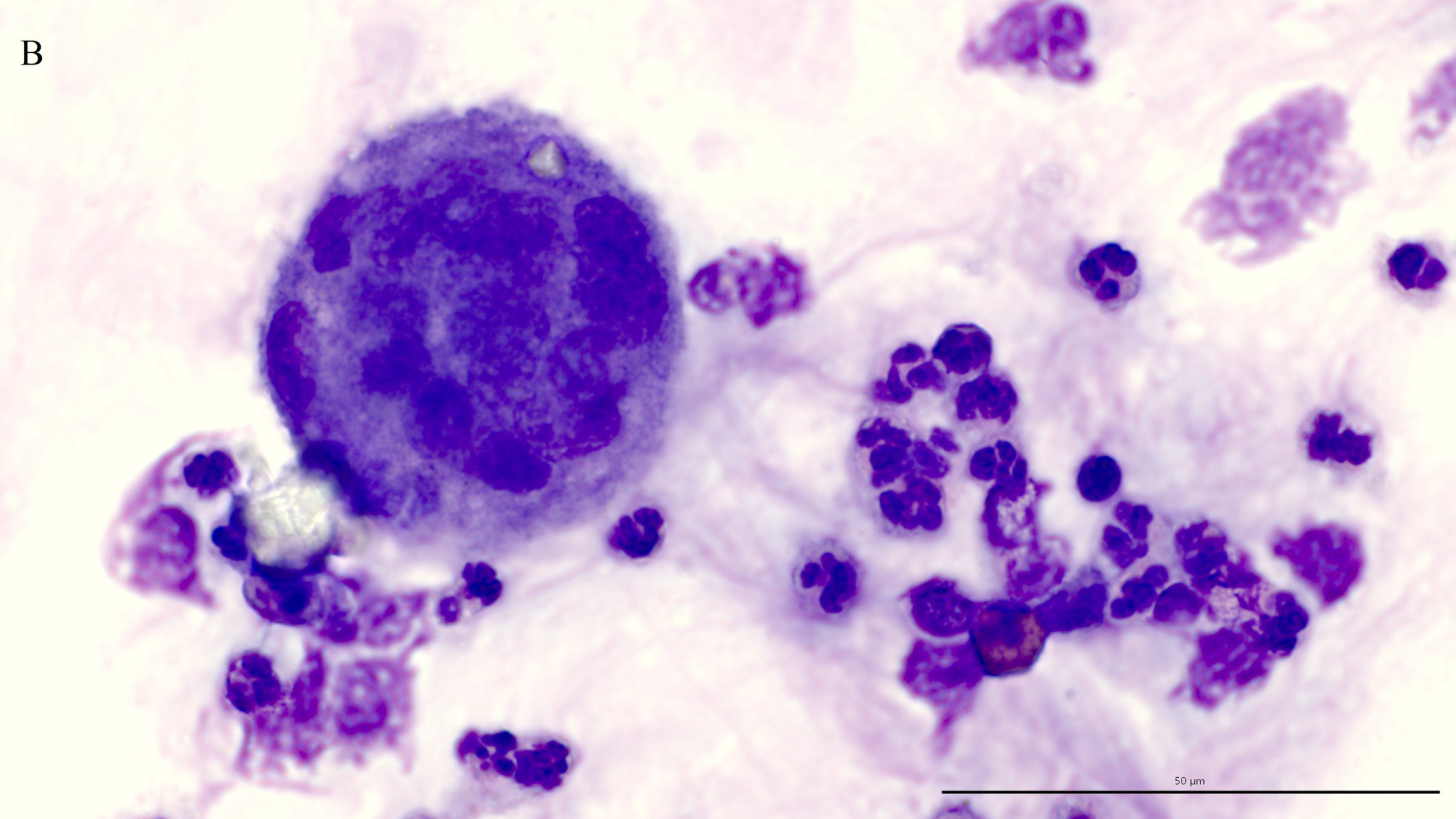
Multinucleated giant cell with ≥ 10 nuclei. Direct smear of tracheal wash stained with modified Wright-Giemsa stain at 1000x oil magnification, bar = 50 μm.

Of the direct smears analyzed, 56% (41/73) had MGCs with ≥ 3 nuclei (Fig 4). Among these, 58% (24/41) originated from asthmatic horses. When considering only MGCs with ≥ 10 nuclei (Fig 5), MGCs were detected in 33% (24/73) of the direct smears, including 42% (10/24) from asthmatic horses.

### Statistical analysis 1 ‐ outcome: Presence of fungal elements in the TW

The results of the univariable analysis for each predictor variable can be found in Table 2. Three predictor variables were selected for entry in the multivariable model based on a P-value < 0.20: BALF neutrophil percentage, feeding type and MGC_3_ or MGC_10_. Although asthma status did not show a significant association with TW fungal elements (P = 0.28), it was retained in the model to prevent the oversight of any potential significant association with the outcome. When considering the predictor variable MGC_3,_ the multivariable statistical model including BALF neutrophil percentage, asthma status and MGC_3_ demonstrated the lowest AIC (Table 4A). Conversely, when considering MGC_10_, the best model included BALF neutrophil percentage, feeding type and MGC_10_ (Table 4B). None of the predictor variables showed a significant association with TW fungi detection.

**Table 4:** A. Best multivariable logistic regression model to predict the presence of TW fungi based on AIC score (including the predictor variable TW MGC_3_). B. Best multivariable logistic regression model to predict the presence of TW fungi based on AIC score (including the predictor variable TW MGC_10_).

Variable	Odds Ratio	CI	*P*-value
BALF neutrophil (%)	1.05	1.00–1.10	0.05
Equine asthma	0.47	0.17–1.31	0.15
TW MGC_3_	2.59	0.96–7.20	0.06
**Variable**	**Odds Ratio**	**CI**	***P*-value**
BALF neutrophil (%)	1.04	1.00–1.10	0.08
Feeding dry hay	2.34	0.78–7.58	0.14
TW MGC_10_	2.88	1.00–8.93	0.06

*TW*, *tracheal wash; MGC*_*3*_, *multinucleated giant cells with ≥ 3 nuclei*.

*TW*, *tracheal wash; MGC*_*10*_, *multinucleated giant cells with ≥ 10 nuclei*.

### Statistical analysis 2 ‐ outcome: Asthma status

The results of the univariable analysis for each predictor variable are presented in Table 3. The presence of MGCs in the TW showed a significant association with asthma status when considering MGC_10_, but not when considering MGC_3_. Although the presence of fungal elements in the TW did not show a significant association with asthma status (P = 0.28), it was retained in the multivariable model to ensure no potential significant association with the outcome was overlooked. For MGC_3_, the multivariable statistical model for asthmatic/control classification including BALF neutrophil percentage, feeding type and tracheal mucus score demonstrated the lowest AIC (Table 5A). When considering MGC_10_, the best multivariable statistical model included the same predictor variables in addition to MGC_10_ (Table 5B). In both models, the tracheal mucus score was the only predictor significantly associated with the asthma status. Horses with a tracheal mucus score ≥ 2 had 3.6 to 4.3 higher odds of being asthmatic, depending on MGC definition (Table 5A and 5B).

**Table 5:** A. Best multivariable logistic regression model to predict the asthma status based on AIC score (including the predictor variable TW MGC_3_). B. Best multivariable logistic regression model to predict the asthma status based on AIC score (including the predictor variable TW MGC_10_).

Variable	Odds Ratio	CI	*P*-value
BALF neutrophil (%)	1.04	0.99–1.10	0.19
Feeding dry hay	0.33	0.09–1.04	0.07
Tracheal mucous score ≥ 2	**3.59**	**1.18–11.94**	**0.03**
**Variable**	**Odds Ratio**	**CI**	***P*-value**
BALF neutrophil (%)	1.04	0.99–1.11	0.14
Feeding dry hay	0.36	0.10–1.16	0.10
Tracheal mucous score ≥ 2	**4.26**	**1.33–15.22**	**0.02**
TW MGC_10_	0.34	0.10–1.06	0.07

*TW*, *tracheal wash; MGC*_*3*_, *multinucleated giant cells with ≥ 3 nuclei*.

*TW*, *tracheal wash; MGC*_*10*_, *multinucleated giant cells with ≥ 10 nuclei*.

## Discussion

This study aimed to investigate the potential association between the equine asthma status and the detection of fungal elements in the TW. Asthmatic horses were matched with control horses from the same barn to account for the effect of environmental exposure. Several potential confounding variables, such as the tracheal mucus score and the presence of MGCs in the TW, were scrutinized. None of the examined variables showed a statistically significant association with the presence of fungi in the TW. Similarly, none of the evaluated variables were associated with asthma status, except for the tracheal mucus score, confirming previous findings [33].

In contrast to the findings of Dauvillier et al. [3], our study did not identify a significant association between the presence of TW fungi and equine asthma. Several factors may contribute to this disparity, including variations in age, breed, activity level, and geographic location across the study populations. Additionally, the definition of asthmatic and control horses was different between the two studies. Dauvillier et al. [3] considered horses as cases (affected with mild-moderate equine asthma) based on BALF cytology results and clinical signs (poor performance and/or coughing), as per the 2016 ACVIM consensus statement [1]. Controls were horses with normal BALF cytology, regardless of clinical signs. In contrast, our study classified horses primarily as asthmatic or controls based on their respiratory history. Moreover, our case population encompassed a broader range of asthma severity, while Dauvillier’s study excluded horses with severe equine asthma. Environmental exposure could also have confounded the former study’s results, as fungal particles in the trachea may reflect the fungal aerosol load in the horse’s environment rather than its asthma status. To address this concern, we matched asthmatic horses with control horses from the same barn and incorporated management factors, such as feeding and bedding type, into the statistical model. This approach aimed to control for potential confounding effects related to environmental exposure, such as variations in barn management or forage quality.

Di Pietro et al. [9] reported a lower fungal particle load in the BALF of stabled asthmatic horses compared to non-asthmatic counterparts. They speculated that this might be linked to lower airway congestion and narrowing in asthmatic horses. Direct comparisons between their findings and ours are challenging due to poor agreement between BALF and TW analysis [8]. In our study, we did not observe a similar negative association, possibly because the TW is more influenced by the aerosol environment than BALF. Furthermore, lower airway narrowing, and congestion are unlikely to impact the fungal load in TW.

Cytological examination revealed the presence of fungal elements in 52% of our TW samples, a notable contrast to the 79% reported by Dauvillier [3] and the 69.4% reported by Lemonnier [8]. Additionally, intracellular fungi were detected in only 34%, a significant deviation from Lemonnier’s 74.4%. The reasons for these discrepancies in prevalence remain unclear, but they could be rooted in differences in the study population and environmental exposure. Notably, the cohorts studied by Dauvillier and Lemonnier primarily consisted of racehorses and sport horses, often recognized for their exercise intolerance and more likely to be stabled. In contrast, our horse population predominantly comprised leisure horses with access to pasture. The lower proportion of intracellular fungal particles in our study likely results from the immediate TW slide preparation following collection. In contrast, Lemonnier et al. [8] processed TW samples within 24 hours, allowing time for in vitro phagocytosis [34].

In their study, Dauvillier et al. [3] documented the presence of germinating spores and branching hyphae in the TW. The number of positive samples was, however, not specified. The identification of proliferating fungi in the airways was considered to be a sign of fungal infection, which could justify the use of antifungal medications in asthmatic horses. In our study, only 3 TW direct smears showed signs of slight active growth (branching hyphae), with 2 of them originating from control horses. Hence, we are confident that none of the horses included in this study had a fungal infection. In Lemonnier’s study [8], branching hyphae were identified in only one TW sample from a clinically healthy horse, further undermining the clinical relevance of this cytological finding.

A recent study demonstrated a significantly higher frequency of MGCs in the BALF of severe asthmatic horses compared to mild-moderate asthmatic and control horses, alongside a negative association with mast cell proportions [24]. Conversely, our study did not identify a significant association between TW MGCs and asthma. This discrepancy may be explained by differences in the type of lower airway inflammation, as our study cohort comprised a mix of mild-moderate and severe asthmatic horses, predominantly exhibiting a neutrophilic inflammatory profile. Alternatively, the discordance could be explained by the known limited agreement between TW and BALF analyses [8]. Although the presence of MGCs in the TW exhibited an association with asthma status in the univariable analysis (P-value < 0.05), this association did not hold significance in the multivariable model. This highlights the intricacy of studying equine asthma, characterized by complex interactions among multiple factors, and emphasizes the importance of considering all potential confounders during data analysis. Interestingly, when considering asthma as the outcome variable, MGC_10_, but not MGC_3_, persisted in the final model. This suggests that the stricter definition of MGCs (≥ 10 nuclei) might be more suitable when studying equine asthma.

The multivariable models with TW fungi as the outcome varied depending on whether the variable MGC3 or MGC10 was included, with one model integrating asthma status as a predictor variable and the other including feeding type. This suggests a degree of interchangeability between these variables, possibly attributable to the reduced likelihood of asthmatic horses being fed dry hay. Interestingly, while equine asthma exhibited an OR < 1 (Table 4A), feeding dry hay yielded an OR > 1, indicating that exposure to dry hay may heighten the likelihood of detecting fungi in TW. This observation aligns with findings from Di Pietro et al. [9], who noted an increase in the number of fungi in the BALF of both control and asthmatic horses after stabling. However, it is important to note that the fungal species found in TW do not necessarily correspond to those detected in hay [8], indicating the existence of diverse sources for fungal particles.

Consistent with previous studies [1,33], we detected a positive association between the tracheal mucus score and the asthma status. On the other hand, the presence of Curschmann’s spirals and the TW scores for neutrophils did not show a significant association with asthma status in the final multivariable model. This supports the notion that TW cytology lacks sensitivity and specificity for diagnosing equine asthma [35].

We acknowledge the limitations of our study, including the relatively small sample size (n = 73). Nonetheless, our study design incorporated case-control matching within the same barn, which should alleviate the impact of the small sample size. This matching strategy ensured that both control and asthmatic horses shared comparable aerosol exposure due to their common environment. Nevertheless, it is important to recognize that certain matched pairs did not receive identical forage or bedding, as some owners made management changes (opting for low-dust alternatives to hay and straw) for asthmatic horses. To address potential bias arising from this imperfect matching, we included relevant management factors, such as feeding and bedding types, in our statistical analyses.

A potential limitation of our study relates to the classification of cases and controls. We did not strictly adhere to the BALF cytology criteria defined in the 2016 IAD consensus statement [1], as these are seldom met in our daily practice. Given that BALF cytology is influenced by dust exposure [36], it proves insufficient to distinguish asthmatic horses from healthy ones in environments with heavy dust load. Therefore, we advocate for a more comprehensive approach, which considers historical, clinical and laboratory parameters, rather than relying solely on BALF cytology to identify asthmatic horses. To facilitate case recruitment, we prioritized the horse’s history and clinical signs, using the HOARSI questionnaire to identify potential asthmatic horses [31]. However, during the presentation of matched control horses at the clinic and upon completing the HOARSI questionnaire, 10 supposedly healthy horses had a HOARSI score exceeding 1. The clinical signs leading to this classification were typically mild, such as a mild nasal discharge and occasional light coughing over the horse’s lifetime. We attribute this observation to the HOARSI questionnaire being more sensitive than specific, making it effective in recognizing severely asthmatic horses but less accurate in distinguishing between healthy and mildly asthmatic horses [37]. Ultimately, our classification of asthmatic and control horses appeared appropriate, as evidenced by significantly different clinical scores, tracheal mucus scores, TW neutrophil semi-quantitative scores and BALF neutrophil percentages between the two groups (Table 1).

In conclusion, our study found no compelling evidence of an association between asthma status and the detection of fungi in the TW of horses when accounting for selected management factors, tracheal mucus score and selected TW and BAL cytological characteristics. This underscores the limited diagnostic utility of TW fungal detection for equine asthma.

## Supporting information

S1 Table Overview of explanatory variables transformation and inclusion status in statistical modelling. (DOCX)

S2 Table Raw data. (XLSX)

## Abbreviations

ABPA: allergic bronchopulmonary aspergillosis
ACVIM: American College of Veterinary Internal Medicine
AIC: Akaike information criterion
BAL: bronchoalveolar lavage
BALF: bronchoalveolar lavage fluid
HOARSI: Horse-Owner Assessed Respiratory Signs Index
IgE: immunoglobulin E
MGC: multinucleated giant cell
TW: tracheal wash
