# Supplementary material for: Association between equine asthma and fungal elements in the tracheal wash: An environment-matched case-control study
Source: PLoS One. 2024 Sep 6;19(9):e0309835. doi: 10.1371/journal.pone.0309835 (PMC11379288; doi:10.1371/journal.pone.0309835)
Supplement: S1 Table — (DOCX) [file pone.0309835.s001.docx]

**S1 Table. Overview of explanatory variables transformation and inclusion status in statistical modelling**

| **Explanatory variable** | **Type (initial)** | **Type (transformed)** | **Considered for statistical modelling with outcome = fungi** | **Considered for statistical modelling with outcome = asthma** | |
| --- | --- | --- | --- | --- | --- |
| Control vs case | Binary  (healthy vs asthmatic) | No transformation | Yes | | No |
| Age | Numeric (continuous) | No transformation | Yes | | Yes |
| Sex | Ordinal (3 levels: mare; gelding; stallion) | Binary (female vs male) | Yes | | Yes |
| Barn | Ordinal (34 levels: 34 different barns) | No transformation | No | | No |
| Feeding type | Ordinal (6 levels: dry hay; straw; wet hay; haylage; steamed hay; hay cubes) | Binary (dry hay and/or straw vs low-dust alternatives) | Yes | | Yes |
| Bedding type | Ordinal (7 levels: straw; sawdust; shavings; hemps; forest floor; straw pellets; rubber mat; other) | Binary (straw vs low-dust alternatives) | Yes | | Yes |
| Tracheal mucus score | Ordinal (6 levels: grades 0-5)^1^ | Binary (grade 0-1 vs grade 2-5) | Yes | | Yes |
| TW overall cellularity | Ordinal (3 levels: 1=rare; 2=moderate; 3=high) | Binary (low-moderate vs high) | No^2^ | | No^2^ |
| TW mucus amount | Ordinal (4 levels: 0=none; 1=rare; 2=moderate; 3=high) | Binary (none-moderate vs high) | No^2^ | | No^2^ |

| Debris on the TW slide | Ordinal (4 levels: 0=none; 1=rare; 2=moderate; 3=high) | Binary (none-moderate vs high) | No^2^ | No^2^ |
| --- | --- | --- | --- | --- |
| TW epithelial cells | Ordinal (4 levels: 0=none; 1=rare; 2=moderate; 3=high) | Binary (none-rare vs moderate-high) | No^2^ | No^2^ |
| TW Curshmann’s spirals | Ordinal (4 levels: 0=none; 1=rare; 2=moderate; 3=high) | Binary (absence vs presence) | Yes | Yes |
| TW fungi | Ordinal (4 levels: 0=none; 1=rare; 2=moderate; 3=high) | Binary (absence vs presence) | No | Yes |
| TW neutrophils | Ordinal (4 levels: 0=none; 1=rare; 2=moderate; 3=high) | Binary (none-rare vs moderate-high) | Yes | Yes |
| TW eosinophils | Ordinal (4 levels: 0=none; 1=rare; 2=moderate; 3=high) | Binary (absence vs presence) | No^3^ | No^3^ |
| TW lymphocytes | Ordinal (4 levels: 0=none; 1=rare; 2=moderate; 3=high) | Binary (absence vs presence) | No^3^ | No^3^ |
| TW mast cells | Ordinal (4 levels: 0=none; 1=rare; 2=moderate; 3=high) | Binary (absence vs presence) | No^3^ | No^3^ |
| TW macrophages | Ordinal (4 levels: 0=none; 1=rare; 2=moderate; 3=high) | Binary (none-moderate vs high) | No^4^ | No^4^ |
| TW MGC_3_  (≥ 3 nuclei) | Ordinal (4 levels: 0=none; 1=rare; 2=moderate; 3=high) | Binary (absence vs presence) | Yes (instead of TW MGCs ≥ 10 nuclei) | Yes (instead of TW MGCs ≥ 10 nuclei) |
| TW MGC_10_  (≥ 10 nuclei) | Ordinal (4 levels: 0=none; 1=rare; 2=moderate; 3=high) | Binary (absence vs presence) | Yes (instead of TW MGCs ≥ 3 nuclei) | Yes (instead of TW MGCs ≥ 3 nuclei) |
| TW bacteria | Binary (absence vs presence) | No transformation | No (only for slide quality assessment) | No (only for slide quality assessment) |
| TW intracellular bacteria | Binary (absence vs presence) | No transformation | No (only for slide quality assessment) | No (only for slide quality assessment) |
| BALF neutrophils percentage | Numeric (continuous) | No transformation | Yes | Yes |

^1^Validated scoring system from Gerber et al., 2004 [17]

^2^ Used for slide quality assessment only

^3^ No or low number of observations per level

^4^ Because macrophage amount is correlated to MGCs, only one of the two variables is considered
